# Meniscus‐Guided Micro‐Printing of Prussian Blue for Smart Electrochromic Display

**DOI:** 10.1002/advs.202205588

**Published:** 2022-11-28

**Authors:** Je Hyeong Kim, Seobin Park, Jinhyuck Ahn, Jaeyeon Pyo, Hayeol Kim, Namhun Kim, Im Doo Jung, Seung Kwon Seol

**Affiliations:** ^1^ Smart 3D Printing Research Team Korea Electrotechnology Research Institute (KERI) Changwon‐si Gyeongsangnam‐do 51543 Republic of Korea; ^2^ Electro‐Functional Materials Engineering University of Science and Technology (UST) Changwon‐si Gyeongsangnam‐do 51543 Republic of Korea; ^3^ Department of Mechanical Engineering Ulsan National Institute of Science and Technology (UNIST) Ulju‐gun Ulsangwang‐yeogsi 44919 Republic of Korea

**Keywords:** augmented reality (AR), electrochromic display, meniscus‐guided printing, micro‐patterning, Prussian blue

## Abstract

Using energy‐saving electrochromic (EC) displays in smart devices for augmented reality makes cost‐effective, easily producible, and efficiently operable devices for specific applications possible. Prussian blue (PB) is a metal‐organic coordinated compound with unique EC properties that limit EC display applications due to the difficulty in PB micro‐patterning. This work presents a novel micro‐printing strategy for PB patterns using localized crystallization of FeFe(CN)_6_ on a substrate confined by the acidic‐ferric‐ferricyanide ink meniscus, followed by thermal reduction at 120 °C, thereby forming PB. Uniform PB patterns can be obtained by manipulating printing parameters, such as the concentration of FeCl_3_·K_3_Fe(CN)_6_, printing speed, and pipette inner diameter. Using a 0.1 M KCl (pH 4) electrolyte, the printed PB pattern is consistently and reversibly converted to Prussian white (CV potential range: −0.2–0.5 V) with 200 CV cycles. The PB‐based EC display with a navigation function integrated into a smart contact lens is able to display directions to a destination to a user by receiving GPS coordinates in real time. This facile method for forming PB micro‐patterns could be used for advanced EC displays and various functional devices.

## Graphical abstract

Meniscus‐guided micro‐printing of Prussian blue (PB) is realized by local crystallization of FeFe(CN)_6_ on a substrate confined with an ink meniscus followed by thermal reduction of Fe^3+^ ions. The printed PB pattern is reversibly converted to Prussian white by conducting cyclic voltammetry in KCl electrolyte. PB‐based electrochromic displays with a navigation function in a smart contact lens are demonstrated.

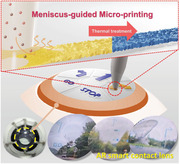

## 1 Introduction

Augmented reality (AR) is a mixture of virtual reality and real life and is rapidly advancing with emergence of the non‐face‐to‐face (untact) culture as the core of the digital economy.^[^ ^1^, ^2^ ^]^ AR is used to produce various types of content that allow users to experience the physical world with digital visual elements, sounds, or other sensory stimuli for education, game, military, and advertising industries.^[^ ^3^, ^4^, ^5^ ^]^

Wearable equipment in the form of glasses is commonly used as a visual tool to implement AR. The representative smart glasses are the Apple Glass, Microsoft Hololens, Google Glass, and Magic Leap, all of which provide augmented or virtual reality on see‐through screens.^[^ ^6^, ^7^, ^8^ ^]^ They can be used for recording, streaming videos, and transmitting data but are not widely used because they are expensive and often bulky.

Smart contact lenses equipped with various functions are attracting attention as an alternative to glasses‐type AR tools. Various studies have been conducted to add different functionalities to contact lenses.^[^ ^9^, ^10^, ^11^, ^12^, ^13^, ^14^, ^15^, ^16^, ^17^, ^18^, ^19^ ^]^ Lee et al. transferred graphene onto a contact lens to prevent electromagnetic waves from entering the eye, thereby preventing eye dehydration.^[^ ^14^ ^]^ Lingley et al. mounted an antenna, IC chip, and LED on a contact lens and wirelessly turned on the LED.^[^ ^15^ ^]^ Google developed an electrical contact lens that monitors glucose levels in the tears of diabetic patients and wirelessly transfers the data to other devices.^[^ ^16^ ^]^ Similarly, Park et al. developed a smart contact lens that integrates LED responses to obtain glucose levels.^[^ ^17^ ^]^ They used a supercapacitor on a contact lens to allow the LED to turn on without the use of an external device.^[^ ^18^ ^]^ Lee et al. integrated a red/near‐infrared (NIR) LED onto a contact lens to treat and prevent diabetic retinopathy.^[^ ^19^ ^]^ These studies focused on developing the integration process of electronic components on contact lenses for wireless control, sensing health states, curing health conditions, and operating single LED that are insufficient for constituting smart contact lenses for AR tools and display implementation. Recently, Mojo Vision, company, demonstrated a prototype of an AR smart contact lens integrated with electronic elements and a micro‐LED display (0.5 mm in diameter/14 000 pixels per inch).^[^ ^20^ ^]^ However, users perceive contact lenses to be inexpensive and disposable, and so, to increase the usability of AR smart contact lenses, it is necessary to develop a cost‐effective, easily producible, and efficiently operable approach for specific applications.

Using electrochromic (EC) materials is a promising approach for simple and inexpensive implementation of displays on contact lenses. A low DC potential applied to a simple cell consisting of a transparent conducting substrate, electrolyte, and EC materials generates electrochemical reduction and oxidation reactions that induce reversible color changes in the EC materials.^[^ ^21^, ^22^, ^23^ ^]^ Prussian blue (PB) is one of the attractive EC materials because of its uniform coloration, fast kinetics, high optical contrast, multiple color states (blue, white, green), environmental friendliness, and cost competitiveness.^[^ ^24^, ^25^, ^26^ ^]^ Kim et al. developed a simple blinking display on a contact lens by employing electroplated PB.^[^ ^27^ ^]^ A PB‐based blinking system capable of displaying Morse code was created using a reversible transition between PB and Prussian white (PW). However, this method has limitations in displaying words or images that are needed for a display on AR smart contact lenses because of the difficulty of micro‐patterning PB on the contact lens.

In this study, we developed a simple strategy for PB micro‐patterning based on meniscus‐guided printing using an acidic‐ferric‐ferricyanide ink composed of FeCl_3_, K_3_Fe(CN)_6_, and HCl. The crystallization of FeFe(CN)_6_ on an indium tin oxide (ITO) substrate was confined to a localized area by an ink meniscus at room temperature. The thermal treatment for 9 s at 120 °C converted the printed FeFe(CN)_6_ pattern to PB (Fe_4_[Fe(CN)_6_]_3_) via the reduction of Fe^3+^ ions. The uniformity and width of the printed PB patterns were successfully controlled by manipulating the printing parameters: the concentration (*C_s_*) of FeCl_3_·K_3_Fe(CN)_6_, printing speed (*ν_p_*), and pipette inner diameter (*ID*). The printed PB pattern was reversibly converted to Prussian white (PW) by conducting cyclic voltammetry in a KCl electrolyte (potential range: −0.2–0.5 V). The EC performance of the printed PB patterns was maintained stably at 0.1 M KCl (pH 4) over 200 cycles. We successfully demonstrated PB‐based EC displays with a navigation function in a smart contact lens by micro‐ printing PB.

## 2 Results and Discussion

**Figure 1:**
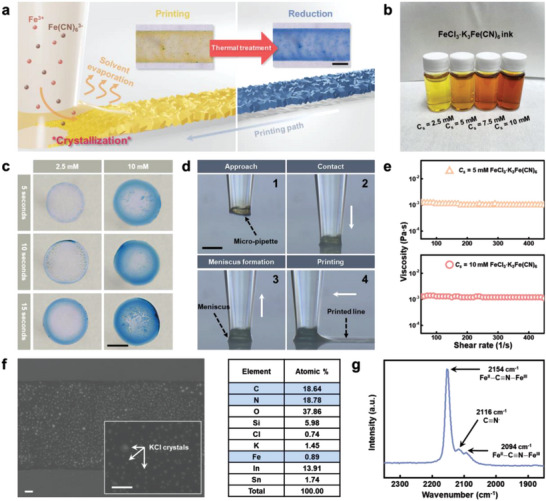
Meniscus‐guided micro‐printing of PB. a) Illustration showing the printing of PB. Crystallization of FeFe(CN)_6_ occurs on the substrate in a region confined by the meniscus, forming the uniform pattern. FeFe(CN)_6_ pattern is converted to the PB (Fe_4_[Fe(CN)_6_]_3_) via thermal reduction. Scale bar is 30 µm. b) Photographs of the inks with different *C_s_* values. c) Optical micrographs of the printed PB dots using a fixing pipette position with a pipette‐substrate gap of 6 µm. The increment of *C_s_* and *T_D_* values contribute to obtain uniformly filled PB dots. Scale bar is 30 µm. d) Real‐time optical micrographs of the meniscus‐guided printing process: approach, contact, meniscus formation, and printing. Scale bar is 30 µm. e) Viscosity of inks with different *C_s_* of 5 and 10 mM as a function of the shear rate. f) FE‐SEM images and EDX element analysis of the PB line. In inset an enlarged image shows that KCl nano‐ and micro‐particles covered the printed line. EDX result indicates that PB is formed well. Scale bar is 5 µm. g) Raman spectroscopy analysis of a PB line. Stretching vibration of cyanide ligands are observed at 2154 and 2094 cm^−1^.

**Figure** **1**a is an illustration of the two‐step PB printing process: (i) meniscus‐guided printing of FeFe(CN)_6_ crystals and (ii) thermal reduction of the printed FeFe(CN)_6_ to obtain PB (Fe_4_[Fe(CN)_6_]_3_). The meniscus of the acidic‐ferric‐ferricyanide ink is formed on the ITO substrate when the ink‐filled micropipette and substrate come in contact. Heterogeneous crystallization of FeFe(CN)_6_ occurs on the substrate within the meniscus via spontaneous reactions of the precursor ions (Fe^3+^ and Fe(CN)^3−^) at room temperature. Simultaneously, the solvent evaporation is occurred at the meniscus surface. When water evaporates from the meniscus, the water molecules and precursor ions move toward the meniscus surface by convective flow, generating a preferential accumulation of the precursor ions in the outer part of the meniscus.^[^ ^28^ ^]^ This phenomenon induces the edge‐enhanced crystallization of FeFe(CN)_6_; this is crucial for controlling the factors that influence the crystallization of FeFe(CN)_6_ in the printing step to obtain uniformly printed PB patterns on a substrate. As will be discussed later, adjustment of the printing parameters, concentration (*C_s_*), printing speed (*ν_p_*), and pipette inner diameter (*ID)*, enables the uniform printed lines to be obtained, as shown in the optical image in Figure 1a. The yellow‐printed FeFe(CN)_6_ line was converted to the blue‐colored PB (Fe_4_[Fe(CN)_6_]_3_) by thermal treatment for 9 s at 120 °C (Figure S1, Supporting Information). Ferric ions (Fe^3+^) were thermally reduced to ferrous ions (Fe^2+^) by receiving electrons from the oxygen groups attached to the ITO substrate and the water molecules in the vacancies and the interstitial sites of the FeFe(CN)_6_ lattice (Figure S2, Supporting Information).^[^ ^29^, ^30^, ^31^ ^]^ The acidic‐ferric‐ferricyanide inks exhibited different colors with respect to *C_s_* of 2.5, 5, 7.5, and 10 mM (Figure 1b).

The impact of different *C_s_* values and deposition times (*T_D_*) at a fixed pipette‐substrate gap of 6 µm on the features of deposited and thermally reduced PB are shown in Figure 1c. Edge‐enhanced deposition of PB was clearly observed for all *T_D_* values at *C_s_* = 2.5 mM. At a higher concentration (*C_s_* = 10 mM) when the *T_D_* increased to 15 s, the dot was almost uniformly filled with PB. These results indicate that a longer crystallization time and larger amount of precursor ions contributed to a denser deposition of FeFe(CN)_6_ in the meniscus. The printing speed (*ν_p_*) is also a key parameter affecting the crystallization time and meniscus shape in real printing processes.

Figure 1d is the real‐time optical micrographs of the meniscus‐guided printing process: approach, contact, meniscus formation, and printing. Micropipette with an *ID* of 30 µm filled with ink (*C_s_* = 5 mM) is approached exquisitely to the ITO substrate. After contact, an ink meniscus is formed between the pipette opening and the substrate by the upward movement of the pipette. During the printing step, the precise horizontal movement (*ν_p_* = 5 µm s^−1^) of the pipette allowed the formation of a uniform FeFe(CN)_6_ line via continuous deposition. The low‐viscosity ink exhibited a Newtonian behavior, indicating that the flow of precursor ions did not cause pipette clogging during printing (Figure 1e and Figure S3, Supporting Information). The Newtonian ink also contributes to form a meniscus constantly onto the planar (even nonplanar) surface by a precise three‐axis control of the pipette, enabling a conformal printing.^[^ ^32^ ^]^

The microstructure and composition of the thermally reduced PB line were analyzed using field emission scanning electron microscopy (FE‐SEM) with energy dispersive X‐ray spectroscopy (EDX) (Figure 1f). In the FE‐SEM image, we can confirm that the PB line is uniformly printed and nano‐ and micro‐particles cover the printed PB line (see inset enlarged image in Figure 1f). These particles are KCl formed as a result of a side reaction during the printing process. The presence of iron (Fe), carbon (C), nitrogen (N), potassium (K), and chloride (Cl) in the printed line is confirmed, indicating formation of PB^[^ ^33^, ^34^ ^]^ and KCl particles. The ratio of oxygen (O) exhibits 37.86% due to the adsorption of moisture from the surrounding environment.^[^ ^35^ ^]^ In general, the oxygen ratio in PB samples can be varied depending on experimental conditions and the environment.^[^ ^33^, ^34^, ^35^, ^36^ ^]^

Raman spectroscopy of the printed PB (Figure 1g) over the wavenumber range of 1900–2300 cm^−1^ shows the stretching vibration of the cyanide ligands coordinated with Fe^2+^ and Fe^3+^. The main peak is the 1*A*_g_ *v*(CN) stretching vibration at 2154 cm^−1^, and the second peak is located at 2094 cm^−1^, corresponding to the *E_g_* mode of the *v*(CN) stretching vibration, clearly indicating that PB was well formed.^[^ ^37^, ^38^ ^]^

**Figure 2:**
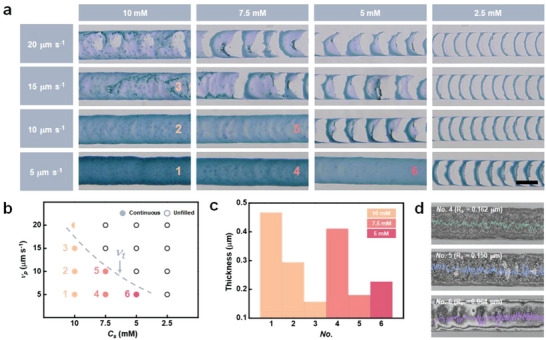
Characterization of printed PB lines as functions of printing parameters. a) Optical micrographs of PB lines printed with an *ID* of 30 µm and different *ν_p_* (5, 10, 15, and 20 µm s^−1^) and *C_s_* (2.5, 5, 7.5, and 10 mM). Well‐printed lines with uniformity are numbered from 1 to 6. Scale bar is 50 µm. b) Diagram displaying the dependence of PB printing on the interplay between *ν_p_* and *C_s_*. Solid circles indicate the continuous deposition under *ν_p_* < *ν_t_*, whereas half solid and hollow circles, indicate the deposition with unfilled parts and the printing failure under *ν_p_* > *ν_t_*. The *ν_t_* increases with *C_s_* as plotted in dotted line. c) Thickness of the numbered lines. d) Surface roughness (*R_a_*) of the line *No*. 4 (*C_s_* = 7.5 mM and *ν_p_* = 5 µm s^−1^), 5 (*C_s_* = 7.5 mM and *ν_p_* = 10 µm s^−1^) and 6 (*C_s_* = 5 mM and *ν_p_* = 5 µm s^−1^).

**Figure** **2**a shows optical micrographs of printed PB lines with different *C_s_* (2.5, 5, 7.5, and 10 mM) and *ν_p_* (5, 10, 15, and 20 µm s^−1^) using an *ID* of 30 µm. At *C_s_* = 10 mM, uniform PB lines were printed at three different *ν_p_* values of 5, 10, and 15 µm s^−1^. This means that there is a sufficient amount of precursor ions and a crystallization time for continuous printing. However, with an increase in *ν_p_* to 20 µm s^−1^, there were unfilled parts in the printed line. A decrease in *C_s_* led to a decrease in the printing speed capable of producing uniform lines, which is defined as the threshold speed (*ν_t_*). As *C_s_* decreased from 7.5 to 5 mM, *ν_t_* also decreased from 10 to 5 µm s^−1^. At *C_s_* = 2.5 mM, discrete lines with numerous unfilled regions were formed in all ranges of *ν_p_* because of the lack of precursor ions in the meniscus. Well‐printed PB lines without unfilled parts are numbered 1 to 6. Figure 2b exhibits the dependence of PB printing on the relationship between *ν_p_* and *C_s_* based on the experimental results presented in Figure 2a. The dotted line shows that *ν_t_* increases with *C_s_*. The solid circles (*No*. 1–6) indicate continuous deposition without unfilled parts under *ν_p_* < *ν_t_*. Meanwhile, half‐solid and hollow circles indicate deposition with unfilled parts and printing failure under *ν_p_* > *ν_t_*, respectively.

The thickness and surface roughness (*R_a_*) of the numbered PB lines were investigated quantitatively using confocal laser scanning microscopy (CLSM) to characterize the morphology of the printed PB line (Figure 2c,d). A lower *ν_p_* and higher *C_s_* led to an increase in the thickness of the printed PB lines. At a *ν_p_* of 5 µm s^−1^, the thickness (≈0.467 µm) of line *No*. 1 (*C_s_* = 10 mM) was larger than that (≈0.227 µm) of line *No*. 6 (*C_s_* = 5 mM) because of the relatively larger supply of precursor ions during the printing process. The larger crystallization time of line *No*. 1 (*ν_p_* = 5 µm s^−1^) gives a larger thickness than that (≈0.157 µm) of line *No*. 3 (*ν_p_* = 15 µm s^−1^) at the same *C_s_* of 10 mM. The *R_a_* of line *No*. 4 (*C_s_* = 7.5 mM and *ν_p_* = 5 µm s^−1^), 5 (*C_s_* = 7.5 mM and *ν_p_* = 10 µm s^−1^) and 6 (*C_s_* = 5 mM and *ν_p_* = 5 µm s^−1^) were 0.162, 0.150, and 0.064 µm, respectively (Figure 2d). The printing condition of line *No*. 6 was selected for the optimized roughness and uniformity of the printed pattern to realize the PB‐based EC AR smart contact lens.

**Figure 3:**
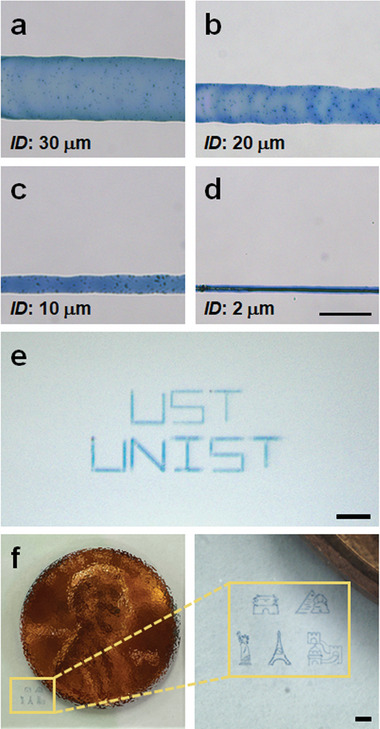
Optical micrographs presenting the dependence of the printed line width on *ID*. a) *ID* = 30 µm (*C_s_* = 5 mM and *ν_p_* = 5 µm s^−1^), b) *ID* = 20 µm (*C_s_* = 5 mM and *ν_p_* = 5 µm s^−1^), c) *ID* = 10 µm (*C_s_* = 5 mM and *ν_p_* = 5 µm s^−1^), d) *ID* = 2 µm (*C_s_* = 1 mM and *ν_p_* = 1 µm s^−1^). Scale bar is 30 µm. e) Printed pattern corresponding to the letters “UST” and “UNIST” in 0.1 M KCl electrolyte (*C_s_* = 5 mM, *ν_p_* = 5 µm s^−1^ and *ID* = 10 µm). Scale bar is 200 µm. f) Printed representative world landmarks (Sungnyemun Gate, Pyramid and Sphinx, Statue of Liberty, Eiffel Tower, and Great Wall of China). Scale bar is 500 µm.

**Figure** **3**a‐d presents that the width of the printed line can be adjusted by controlling the meniscus size using an *ID* of 30, 20, 10, or 2 µm which are shown in Figure S4, Supporting Information. The line width decreased from 61.3 to 17.3 µm as *ID* decreased from 30 to 10 µm (Figure 3a‐c). The lines were printed using the printing conditions of *No*. 6 above (*C_s_* = 5 mM and *ν_p_* = 5 µm s^−1^). No pipette clogging was observed with decreasing *ID* before 2 µm, where clogging was caused by the excessive crystallization of FeFe(CN)_6_ because of the relatively larger solvent evaporation from the small meniscus with a high volume‐to‐surface area. The excessive crystallization in the meniscus was suppressed by reducing the *C_s_* and *ν_p_* values (*C_s_* = 1 mM and *ν_p_* = 1 µm s^−1^) to solve this issue. Consequently, a uniform PB line with 7.2 µm width was successfully printed, as shown in Figure 3d. Figure 3e,f shows the printed micro‐patterns of PB: the pattern corresponding to the letters “UST” and “UNIST” (Figure 3e) and the representative world landmarks (Figure 3f). All the patterns were printed with an *ID* of 10–20 µm and the conditions used in *No*. 6 were applied. Compared to the US penny, the printed landmarks with line width of 30 µm in Figure 3f were ≈20 times smaller. Reducing the printable width to a sub‐micrometer is underway through investigations of *ID*, *C_s_*, *ν_p_*, humidity, and ink composition. In this study, we focused on the implementation of EC displays utilizing printed PB patterns with widths of tens of micrometers.

**Figure 4:**
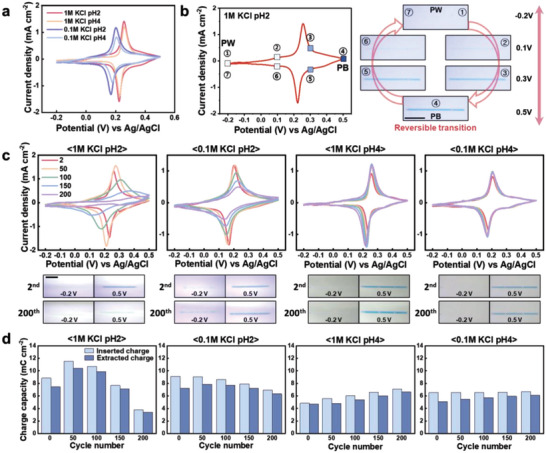
Electrochemical and optical characterization of printed PB line. a) Cyclic voltammogram of PB lines in 1 M KCl (pH 2 and 4) and 0.1 M KCl (pH 2 and 4) with a scan rate of 20 mV s^−1^. b) Cyclic voltammogram and optical micrographs of PB line at different potentials (−0.2, 0.1, 0.3, and 0.5 V) in 1 M KCl (pH 2). Scale bar is 500 µm. c) Performance of PB line in 1 M KCl (pH 2 and 4) and 0.1 M KCl (pH 2 and 4). Scale bar is 500 µm. d) Diagram of charge capacity (inserted and extracted) with respect to cycle number, calculated based on Figure 4c.

The properties of the printed PB pattern, such as uniform coloration, stability, and capacitance, were investigated via cyclic voltammetry (CV). For the CV tests, PB lines with a length of 1500 µm were printed with the conditions of *No*. 6 and an *ID* of 30 µm onto an ITO glass. An ITO substrate without PB patterns was used for CV testing to investigate the influence of the blank ITO on the CV results that were shown to be effectively negligible, as shown in Figure S5, Supporting Information. **Figure** **4**a shows the CV results measured in the 2^nd^ cycle in four different electrolyte conditions of 0.1 M KCl (pH 2 and 4) and 1 M KCl (pH 2 and 4) in the potential range of −0.2 and 0.5 V versus Ag/AgCl (scan rate of 20 mV s^−1^). All the CV curves exhibited clear redox peaks that indicate the insertion and extraction of K^+^ ions. As the KCl concentration in the electrolyte increased from 0.1 M to 1 M, the potential of the redox peaks shifted to a higher value because of the increase in reactive (insertion/extraction) K^+^ ions as oxidizers in the redox reaction.^[^ ^39^ ^]^ In addition, the decrease in the pH of the electrolyte led to an increase in the reactivity of the electrolyte, contributing to an increase in the peak current density. These results are consistent with previously reported results for deposited PB films.^[^ ^40^, ^41^ ^]^

In Figure 4b, The printed PB line was reversibly converted to Prussian white (PW) at a potential range of −0.2 and 0.5 V versus Ag/AgCl in 1 M KCl (pH 2) electrolyte (Figure 4b and Video S1, Supporting Information). Further application of a positive potential resulted in the oxidation of PB to Prussian green (PG),^[^ ^42^ ^]^ which is a soluble and unstable phase (Figure S6 and Video S2, Supporting Information). The number indicates the scanning sequence in the CV test. The numbered optical images present the color change of the printed pattern at the same numbered condition. At −0.2 V, the line color was completely white (PW) (①). As the potential increased to 0.5 V, the color gradually changed to blue, indicating the transition from PW to PB (④). The return of the potential to −0.2 V induced the successful transition of PB (④) back to PW (⑦). The electrochromism of the PB‐based display, displayed on the CIE 1931 color space, shows a distinct contrast along the blue‐hue line with a change in voltage (Figure S7, Supporting Information). This clearly shows that the printed PB line has an excellent EC coloration.

Figure 4c presents the CV curves (the 2^nd^, 50^th^, 100^th^, 150^th^, and 200^th^ cycles) of the PB line in 1 M KCl (pH 2 and 4) and 0.1 M KCl (pH 2 and 4) electrolytes. As the cycle number increased in 1 M KCl (pH 2) electrolyte, the area of the CV curves and the magnitude of the redox peak rapidly reduced, indicating that the lattice structure of PB could be easily destroyed by the K^+^ ions as the main charge compensator and the excessive H^+^ ions during their insertion (extraction) into (from) the printed PB line.^[^ ^40^ ^]^ Consequently, the color of the printed PB line faded after the 200^th^ cycle. In the 0.1 M KCl (pH 2) electrolyte, the reduced KCl concentration caused the printed line to exhibit a slower degradation behavior during the 200 cycles because of the reduction in the concentration of K^+^ ions led to a reduction in the amount of degradation in the printed line. The CV curves in the right two graphs of Figure 4c show the effect of pH on the reversible behavior of the line. In the 0.1 M KCl (pH 4) electrolyte, the area of the CV curves and the magnitude of the redox peak were constantly maintained over the 200 cycles, resulting in a reasonable reversibility of the printed PB line (Video S3, Supporting Information). It is reported that acidified KCl solution generally improves the durability of PB, but excess H^+^ ions rather destroy the lattice structure of PB.^[^ ^40^, ^43^ ^]^ The optimal amount of H^+^ ions in the electrolyte of pH 4 prevents damage to the lattice structure of PB, thereby showing stable repeatability and color change. The stable EC properties of the printed PB in the electrolyte with a lower KCl concentration and a higher pH are due to the characteristics of our printed pattern with a small thickness and a high volume‐to‐active surface area.

Figure 4d shows the extracted and inserted charge capacities of the printed lines as a function of the CV cycle number, calculated based on Figure 4c. The variation in charge capacity was minimal in a 0.1 M KCl (pH 4) electrolyte, indicating that the performance of the PB pattern is sustainable during CV cycles. The relatively low current density in 0.1 M KCl (pH 4) electrolyte is sufficient to induce the electrochromism of the printed PB pattern and maintain its performance. An electrolyte including 0.1 M KCl (pH 4) was selected for implementation of the PB‐based EC display in the smart contact lens by using the printed PB micro‐patterns.

**Figure 5:**
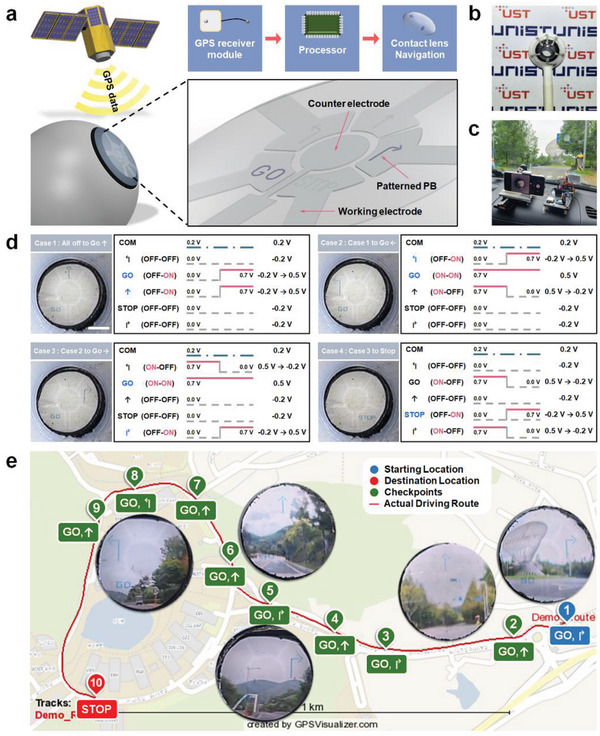
EC display for navigation system embedded in a contact lens. a) Schematic of the EC navigation system integrated with a smart contact lens consisting of GPS receiver module, Arduino UNO as a processor, and PB display. b) Photograph of contact lens placed on the 3D printed replica eyeball. c) Camera setup of the navigation system on the dashboard of a car. d) Driving schemes updating the direction signal: (1–4) images show the four cases of operational principles used in the navigation system. Based on 0.2 V applied to the common pin, 0 V (off‐state) and 0.7 V (on‐state) are applied alternately in 5 WEs, and operating voltages with relative voltages of −0.2 V and 0.5 V are obtained (From the figure reads left to right: the name of 6 pins used in the system, their on–off status, the applied voltage, and relative voltage). Scale bar is 2 mm. e) Actual GPS coordinates route operated by the navigation system, 10 checkpoints, and pictures of the direction signal at 5 checkpoints. (Points 1, 2, 5, 6, and 8 from the right).

**Figure** **5**a presents a schematic of the PB‐based EC display with a navigation function in an AR smart contact lens that shows directions to the destination to a user on the EC display by receiving GPS coordinates in real time. Direction arrows (straight, left, and right) and signs (GO and STOP) appear based on the latitude and longitude information from the GPS by programmed logic to control the applied voltage between a counter electrode (CE) and five working electrodes (WE). As shown in Figure 5b, the EC display‐integrated hydrogel contact lens was placed on a 3D‐printed replica eyeball. The electrolyte of 0.1 M KCl (pH 4) electrolyte was placed in the inner space between the EC display and hydrogel lens. Images of the PB patterns and scenery were obtained together through a hole in the pupil of the replica eyeball. An image‐capturing module was established to obtain an in situ visual image during the operation of the smart contact lens integrated with the navigation system (Figure 5c).

Figure 5d shows the driving schemes of the four types of direction indication methods used in the navigation systems. The first data (Case 1) shows how to turn on the forwards arrow and the “GO” pattern when all segments are in the OFF state. While the applied voltage of 0.5 V caused the up arrow and the “GO” pattern to be blue, −0.2 V is applied continuously to the OFF‐state segments. When switching from Case 1 to Case 2, the applied voltage at the “GO” pattern is maintained at 0.5 V. The forward arrow turns OFF‐state when the applied voltage is changed from 0.5 to −0.2 V. At the same time, the color of the leftwards arrow is changed to blue using 0.5 V. For the remaining OFF‐state patterns, the applied voltage is kept in constant at −0.2 V. A similar operation is used to realize the EC navigation display in cases of 3 and 4.

The transition between PB and PW is an important indicator to evaluate the response time of the display. The response time was measured by modulating duration values (0.1 s, 0.25 s, 0.5 s, and 0.75 s) at the alternating potential of −0.2 V and 0.5 V in a chronoamperometry test (Figure S8, Supporting Information). The PB‐based EC display exhibits stable blinking even in 0.1 s duration condition, making it possible to quickly deliver information to users (Video S4, Supporting Information). In our demonstration, considering the user's visibility, the on/off time is set to 1 s and 2 s, respectively (Video S5, Supporting Information).

Figure 5e presents the actual driving route, 10 checkpoints, and images of direction indications at five points. Five photographs were captured when the on‐state in the blinking mode operated with a 2 s on‐state and 1 s off‐state. While the vehicle was moving, the changing scenery and direction indicator signals matched well and were presented clearly to the user. A total of 83 coordinate sections were passed from the starting point to the destination point, and the EC display of the contact lens showed stable color‐change repeatability without noticeable performance degradation (Video S6, Supporting Information). The AR‐integrated smart contact lens successfully delivered directional information to users in a real‐time changing environment, which is sufficient for use as an advanced display device that can implement AR.

## 3 Conclusion

We report a simple and effective printing strategy to produce micro‐patterns of PB using the meniscus‐guided printing of an acidic‐ferric‐ferricyanide ink composed of FeCl_3_, K_3_Fe(CN)_6_, and HCl. The ink exhibited a Newtonian behavior that was supplied continuously to the substrate without the clogging of the pipette during printing. PB patterning was realized by the localized crystallization of FeFe(CN)_6_ on the substrate confined by the ink meniscus and thermal reduction of the crystallized FeFe(CN)_6_. After the thermal treatment for 9 s at 120 °C, the printed FeFe(CN)_6_ pattern was converted to PB (Fe_4_[Fe(CN)_6_]_3_) by the reduction of Fe^3+^ ions. A sufficient amount of precursor ions and enough crystallization time to obtain well‐printed PB patterns without unfilled parts were achieved by modulating printing parameters such as the concentration (*C_s_*) of FeCl_3_·K_3_Fe(CN)_6_, printing speed (*ν_p_*), and pipette innter diameter (*ID)*. The minimum width of the printed pattern could be easily adjusted by controlling the meniscus size with *ID*, resulting in the formation of 7.2 µm a width line at an *ID* of 2 µm. Reducing the printable width to a sub‐micrometer requires further investigation into *ID*, *C_s_*, *ν_p_*, humidity, and ink composition. The printed PB pattern in the KCl electrolyte was reversibly modulated to PW (CV potential range: −0.2–0.5 V) and its transition behavior was stably maintained during the 200 CV cycles. PB‐based EC displays in a smart contact lens with a navigation function were successfully demonstrated. The device was able to display directions to the destination to the user on the EC display by receiving GPS coordinates in real time. In previous studies, we successfully printed graphene, CNT, and metallic micro‐patterns or 3D micro‐architectures on flexible materials using the meniscus‐guided printing approach.^[^ ^32^, ^44^, ^45^, ^46^, ^47^, ^48^ ^]^ Although thin glass ITO was used for the EC display in this study, it can be further developed as a method of patterning transparent electrodes such as graphene on flexible materials and printing EC materials. We believe that our novel strategy will serve as an attractive method for realizing PB‐based EC displays as well as diverse functional devices with micro PB patterns.

## 4 Experimental Section

### Preparation and Characterization of Ink

PB precursor ink was prepared by mixing potassium hexacyanoferrate(III) (K_3_Fe(CN)_6_, 99%, Sigma‐Aldrich), Iron(III) chloride (FeCl_3_, 97%, Sigma‐Aldrich), and 0.104 g of hydrochloric acid (HCl, 35%, Daejung Chemicals & Metals Co., LTD) in water. The molar ratio of K_3_Fe(CN)_6_ to FeCl_3_ in the ink was 1:1. The rheological properties of the inks were characterized using a rheometer (MCR102, Anton Paar). A strain sweep was conducted from 10 to 500 s^−1^ to measure ink viscosity at varying shear rates. A stress sweep at a constant frequency of 1 Hz was performed to record the variations in the storage and loss moduli as functions of the sweep stress.

### Meniscus‐Guided Printing

PB micro‐patterns were printed using a meniscus‐guided printing approach. Glass micropipettes with *ID*s of 2, 10, 20, and 30 µm were obtained using a pipette puller (P‐2000, Sutter Instruments). The ink was filled through the back of the micropipette using capillary forces with no applied pressure. Indium tin oxide‐coated glass with a resistance of 4–6 Ω cm^−2^ (AMG Tech) was used as the printing substrate. During the printing process, the pipette motion corresponding to the printed paths was determined using parameterized G‐code scripts and controlled by three‐axis stepping motors with 250 nm positioning accuracy. The nozzle motion corresponds to the printed paths. The printing process was observed in situ using a high‐resolution monitoring system consisting of an optical objective lens (50 ×, Mitutoyo) and complementary metal oxide semiconductor camera (BFS‐U3_200S6C, Flir). The printed patterns were treated thermally on a hot plate for 9 s at 120°C for reduction.

### Characterization of Printed PB

The microscopic characteristics of the printed PB were analyzed using FE‐SEM (S‐4800, HITACHI), optical microscopy (BX53M, Olympus), and confocal laser scanning microscopy (CLSM) (VK9700K, KEYENCE). The elemental analysis of the printed PB was conducted using EDX (EX‐470 X‐Max150). Raman analysis was performed using a WITEC Alpha 300R with a 0.06 mW 523 nm excitation laser, which was used to avoid damage to the specimen. Raman spectra were recorded in the spectral range between 120 and 3200 cm^−1^ and focused on the triple bond between the carbon and nitrogen bands at 1900 and 2400 cm^−1^. The acquisition time was set at 60 s and accumulated three times. The electrochemical properties were investigated by cyclic voltammetry (CV) using an AUTOLAB PGSTAT204 potentiostat/galvanostat at room temperature with an electrolyte prepared by mixing potassium chloride (99.0%, SamChun) and hydrochloric acid (90.0%, SamChun) in water. A Pt sheet electrode and Ag/AgCl saturated with 3 M KCl were used as the counter and reference electrodes, respectively. CV analysis was performed at a scan rate of 20 mV s^−1^ in the range of −0.2–0.5 V. The response time of the PB‐based EC display was measured by modulating duration values (0.1 s, 0.25 s, 0.5 s, and 0.75 s) at the alternating potential of −0.2 V and 0.5 V in a chronoamperometry test. The mean RGB data of the printed PB patterns were obtained from the video of the CV test taken by a CCD camera (BASLER, a2A4504‐18ucBAS).

### Implementation of PB Electrochromic Display in Contact Lenses

A 3D plastic template for manufacturing a silicon mold of hydrogel contact lenses was fabricated via stereolithography (SLA) with an acrylonitrile butadiene styrene (ABS) resin. The concave and convex silicon molds were fabricated by pouring silicon rubber (Smooth‐on, Ecoflex 0030) into a plastic template.^[^ ^14^ ^]^ 5 ml of 2‐Hydroxyethyl methacrylate (HEMA, 99.0%, Sigma Aldrich) was mixed with 0.085 g of ethylene glycol dimethacrylate (EGDMA, 98.0%, Sigma Aldrich) as the crosslinking agent. Subsequently, 3 ml of deionized (DI) water and the UV initiator, 0.085 g of 2‐Hydroxy‐2‐methylpropiophenone (97.0%, Sigma Aldrich), were added and then stirred.^[^ ^13^ ^]^ The prepared HEMA solution was coated onto the surface of the convex mold by pushing the convex mold into a concave mold containing the HEMA solution. A soft hydrogel contact lens with a curvature radius of 8.6 mm and a diameter of 12.5 mm was fabricated by UV polymerization of the HEMA solution coated on the convex mold. Two sets of 1 min exposure to UV light (400 W, DY‐max) with a 1 min rest between were conducted. The hydrogel contact lens was removed from the mold using an ultrasonic cleaner, then the lens was cut to a diameter of 12.5 mm. ITO‐coated glass (diameter: 9 mm, thickness of 0.7 mm, and resistance: 4–6 Ω cm^−2^) was patterned by a laser (AMG tech), resulting in the formation of six segments (one counter electrode and five working electrodes in a two‐electrode electrochemical cell). The ITO electrodes were arranged within a circular pattern area with a diameter of 5 mm, which is the average size of a human pupil. The areas extending outward from the electrodes were designed to avoid interference with the field of vision, as they correspond to the iris area of the human eye. Within the five working electrodes, three direction arrows and two letters (“GO” and “STOP”) were printed by the meniscus‐guided printing of PB. The circular area corresponding to the counter electrode was placed at the center of the lens for viewing. Enamel wires were attached to the edge of the ITO electrodes using silver paste to increase conductivity. The PB EC display was integrated with a hydrogel lens, and a 0.1 M KCl (pH 4.0) electrolyte was injected between them.

### Navigation Control System

The Arduino UNO and GPS module (NEO‐6M) were used to implement the navigation system. The real‐time latitude and longitude data obtained from the GPS module recognized the current location of the user through an Arduino UNO processor. Using lab‐made code the coordinates of 10 checkpoints were predesignated to show the direction we wanted on the EC display at the specific coordinates; in consideration of the GPS error, a recognition radius was set at ≈16 m. Whenever passing through each of the 10 checkpoints, it changed to the next direction indicator, and while moving between the two checkpoints, it was set to continue to show the directions of the previous checkpoint. A signal output from the Arduino was used to drive the EC display. A two‐electrode EC system using Arduino was used to implement the PB state for an applied voltage of 0.5 V and the PW state for an applied voltage of −0.2 V, which is the voltage range of the color change of PB used in the CV test. The voltages from the Arduino's 6 digital pins were decreased from 5 to 0.2 V for the COM pin and 0.7 V for the remaining 5 pins using potentiometers (200 Ω), and each was connected to 6 electrodes on the EC display. The COM pin continuously outputs a 0.2 V voltage, and at the same time, 0.0 V and 0.7 V were output alternately according to the signal from the 5 digital pins for the WE. As a result, the relative voltages between CE and WEs were obtained as −0.2 V and 0.5 V. To turn off (transparent state) the specific PB patterns, the output voltage of the WE is set to 0.0 V, thereby the relative voltage of −0.2 V is applied to the PB pattern. Conversely, to turn on (blue state) the specific PB patterns, 0.7 V was applied to the WE, thereby the relative voltage of 0.5 V is applied to the PB patterns. The demonstration videos of the navigation system were captured by Galaxy S22+ with a 5.0 × zoom. The smartphone camera observed the contact lens surface to capture the color change of the printed PB, and an external macro lens (APEXEL, 10x) was placed in front of the contact lens to simultaneously bring the landscape into focus. The image‐capturing module and the Arduino with the GPS module were set on the car dashboard.

## Conflict of Interest

The authors declare no conflict of interest.

## Supporting information

Supporting Information

Supplemental Video 1

Supplemental Video 2

Supplemental Video 3

Supplemental Video 4

Supplemental Video 5

Supplemental Video 6

## Data Availability

The data that support the findings of this study are available from the corresponding author upon reasonable request.
