# Supplementary material for: Meniscus‐Guided Micro‐Printing of Prussian Blue for Smart Electrochromic Display
Source: Adv Sci (Weinh). 2022 Nov 28;10(3):2205588. doi: 10.1002/advs.202205588 (PMC9875632; doi:10.1002/advs.202205588)
Supplement: Supplementary file 1 — Supporting Information [file ADVS-10-2205588-s007.pdf]

Supporting Information

Meniscus-guided Micro-printing of Prussian Blue for Smart Electrochromic Display

Je Hyeong Kim, Seobin Park, Jinhyuck Ahn, Jaeyeon Pyo, Hayeol Kim, Namhun Kim, Im Doo Jung, and Seung Kwon Seol**

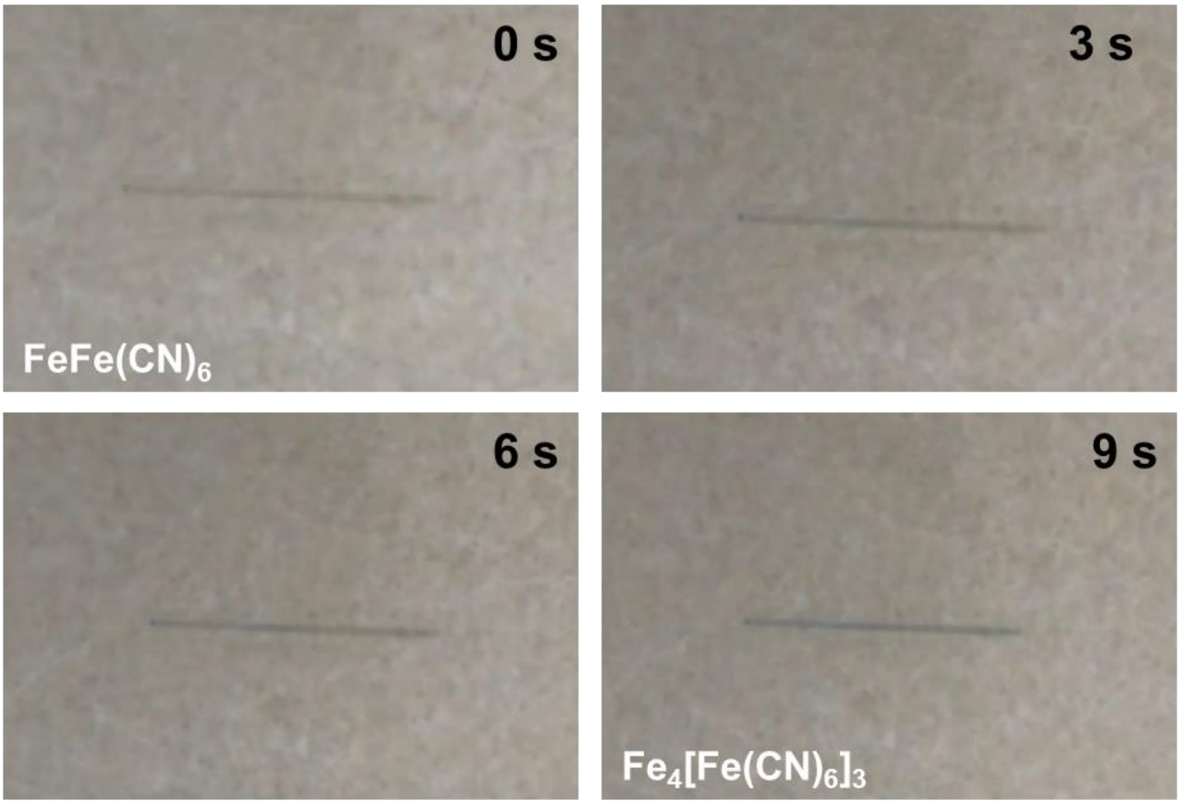

Figure S1. The yellow-printed $FeFe(CN)_6$ line with (C_s = 5mM, v_p = 5 um s^{-1}, *ID* of 30 μm) was converted to the blue colored PB ($Fe_4[Fe(CN)_6]_3$) in 9 s at 120 °C.

a

$$Fe^{3+} + [Fe^{(III)}(CN)_6]^{3-} + 2H_2O \rightarrow Fe^{2+} + [Fe^{(II)}(CN)_5]^{3-} + NH_4^{+} + CO_2 \quad (1)$$

$$Fe^{2+} + [Fe^{(III)}(CN)_6]^{3-} \rightarrow Fe^{3+} + [Fe^{(II)}(CN)_6]^{4-} \quad (2)$$

$$4Fe^{3+} + 3[Fe^{(II)}(CN)_6]^{4-} \rightarrow Fe^{(III)}_4[Fe^{(II)}(CN)_6]_3 \text{ (Prussian blue)} \quad (3)$$

b

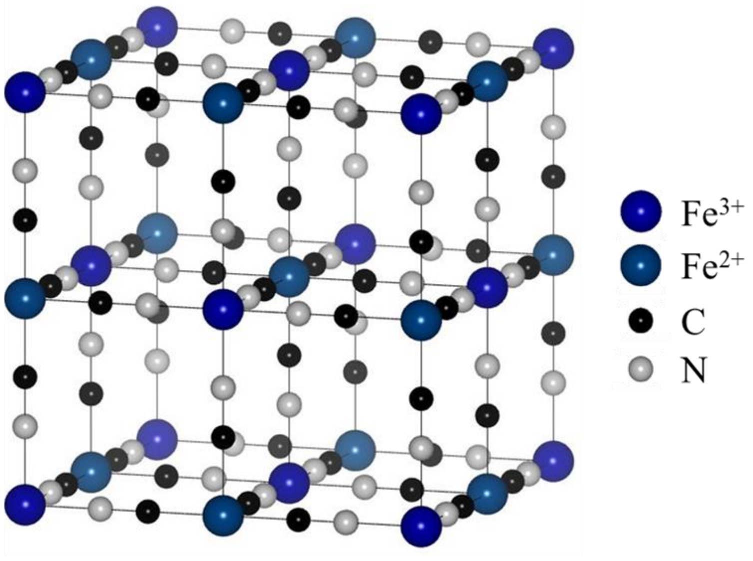

<Prussian blue>

Figure S2. a) Mechanism of the printed FeFe(CN)$_6$ transform to PB (Fe$_4$[Fe(CN)$_6$]$_3$) during the thermal treatment process. b) Atomic structure of PB consisting of Fe^{II}-C-N-Fe^{III} sequences.

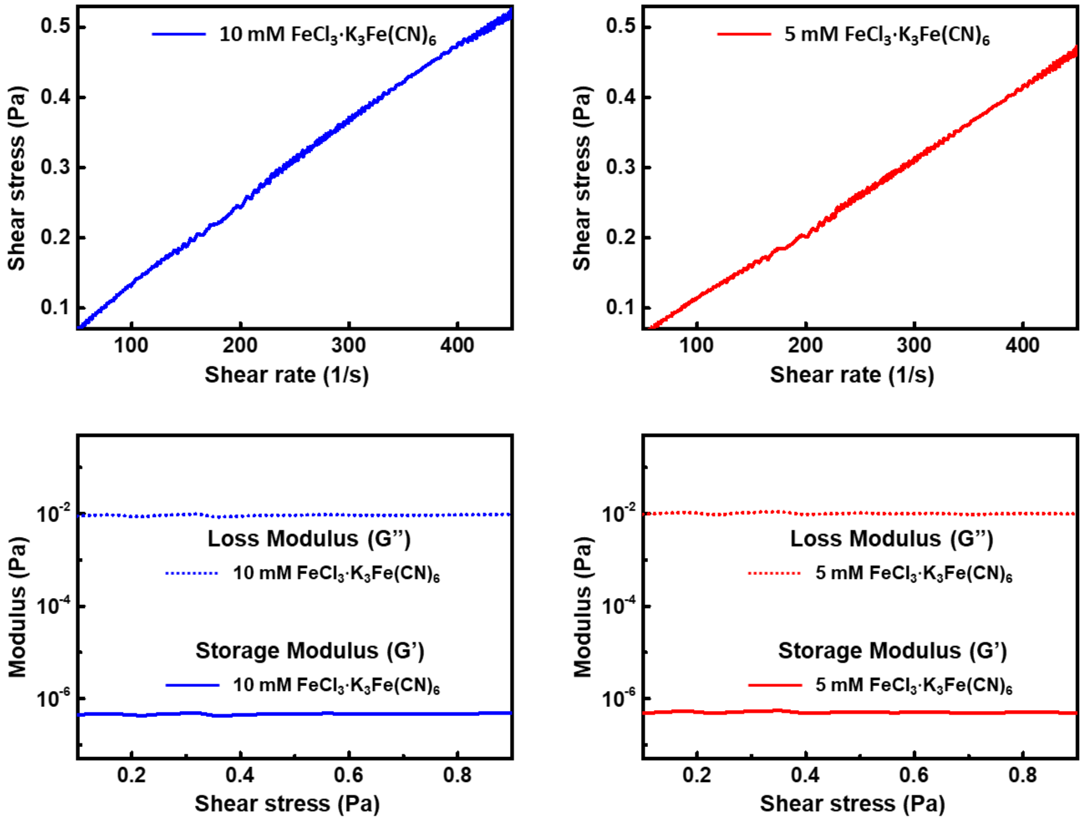

Figure S3. Rheological properties of acidic-ferric-ferricyanide ink composed of $FeCl_3$, $K_3Fe(CN)_6$, and HCl. The inks exhibit a Newtonian fluid behavior in both C_s of 2.5 mM and 10 mM, enabling to form $FeFe(CN)_6$ micro-patterns without pipette clogging during printing process.

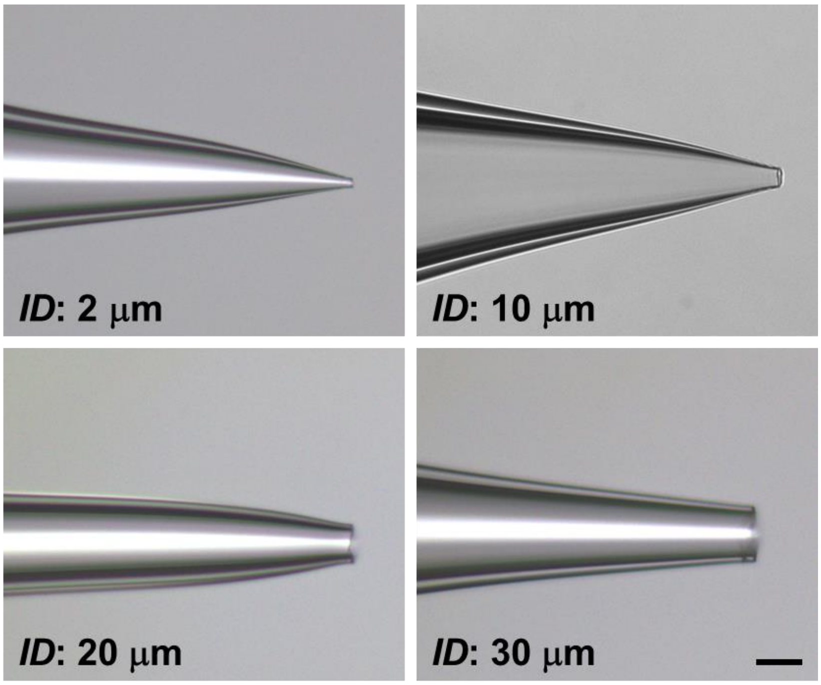

Figure S4. Optical images of micropipettes (*ID* = 2, 10, 20, and 30 μm). The size of ink meniscus can be controlled with *ID* of micropipettes during the printing process. Scale bar is 30 μm.

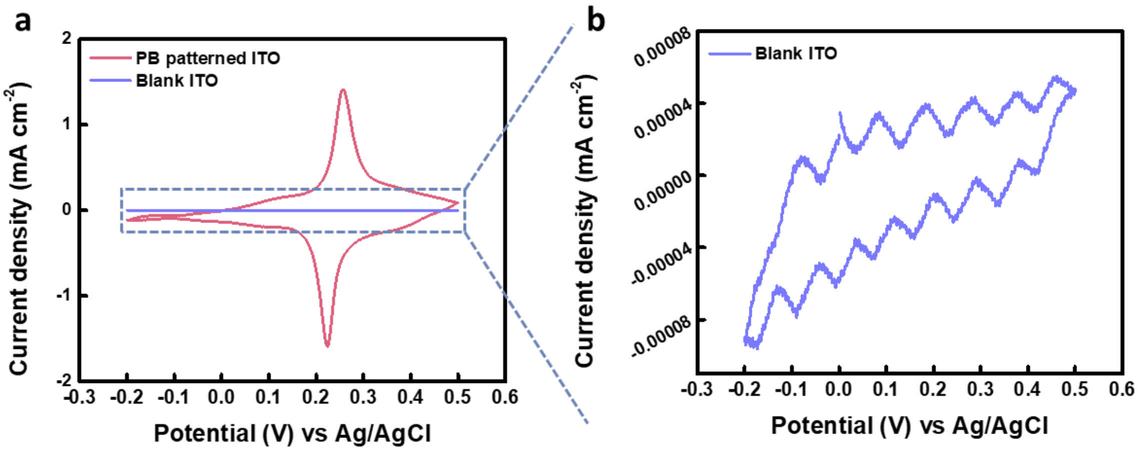

Figure S5. Cyclic voltammetry (CV) curves of the PB patterned ITO and the blank ITO in 1 M KCl (pH 2) electrolyte with scan rate 20 mV s^{-1}. a) Comparison of CV result between the PB patterned ITO and the blank ITO. b) CV curve of the blank ITO.

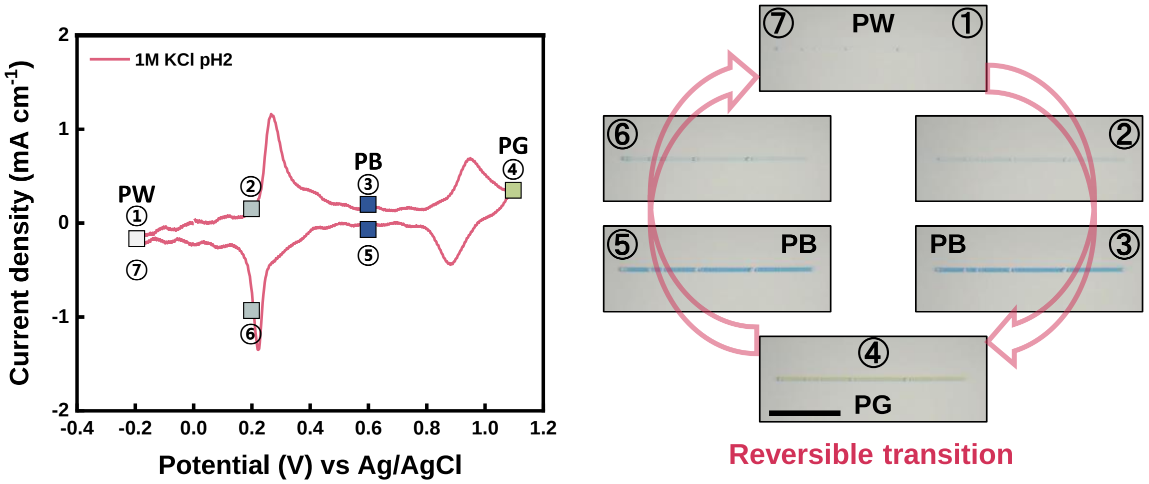

Figure S6. Cyclic voltammogram and optical micrographs of PB line at different potentials (-0.2, 0.2, 0.6, and 1.1 V) in 1 M KCl (pH 2) with scan rate 20 mV s^{-1}. The printed PB line was reversibly converted from PW to PG at a potential range of -0.2 and 1.1 V *vs.* Ag/AgCl in 1 M KCl (pH2) electrolyte. Scale bar is 500 μm.

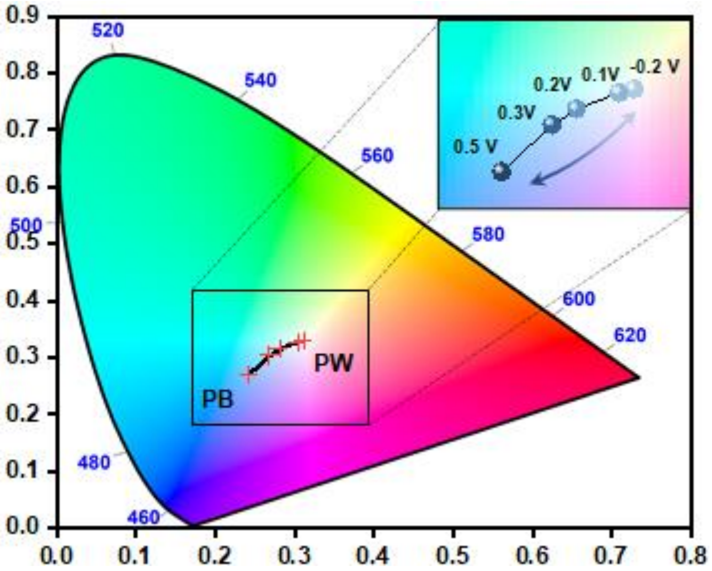

Figure S7. Chromaticity coordinate plots of PB-based EC display in CIE 1931 color space. As the applied voltage is changed from 0.5 V (-0.2 V) to -0.2 V (0.5 V), the color of printed pattern is changed clearly from the blue (white) area to the white (blue) area along the iso-hue line.

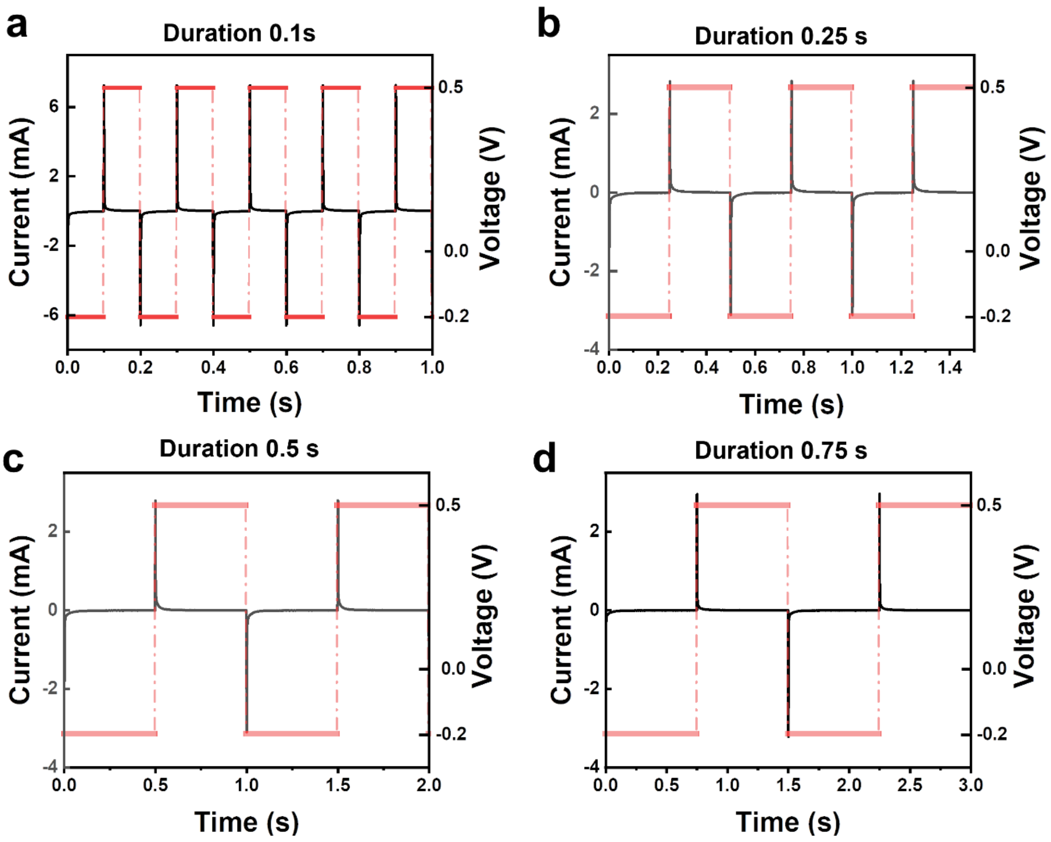

Figure S8. Chronoamperogram of the printed PB pattern measured by modulating duration values at the alternating potential of -0.2 V and 0.5 V in 0.1 M KCl pH 4 electrolyte (vs Ag/AgCl). a) Duration: 0.1 s. b) Duration: 0.25 s. c) Duration: 0.5 s. Duration: 0.75 s.

Video S1. Reversible conversion of Prussian blue (PB) to Prussian white (PW) at a potential range of -0.2 and 0.5 V (*vs.* Ag/AgCl) with a scan rate of 20mV s^{-1} in 1 M KCl (pH 2) electrolyte. The video is played at 2x speed.

Video S2. Reversible conversion of Prussian blue (PB) to Prussian green (PG) at a potential range of -0.2 and 1.1 V (*vs.* Ag/AgCl) with a scan rate of 20mV s^{-1} in 1 M KCl (pH 2) electrolyte. The video is played at 2x speed.

Video S3. The durability of the printed PB line up to the 250 cycles of the CV test (potential range: -0.2 and 0.5 V (*vs.* Ag/AgCl) with a scan rate of 20mV s^{-1} and electrolyte: 0.1 M KCl (pH 4)). The video is played at 5x speed.

Video S4. The measurement of the response time of the PB-based EC display. The duration time at the alternating potential of -0.2 V and 0.5 V is modulated to 0.1 s, 0.25 s, 0.5 s, and 0.75 s. The video is played at 1x speed.

Video S5. Operation of the EC navigation display embedded in a contact lens. In the blinking mode, the PB pattern turns on for 3s and turns off for 1s to switch the signal. In the non-blinking mode, the PB pattern is converted to the next signal without an interval to turn off. The video is played at 1x speed.

Video S6. Operation of the EC navigation display along the actual GPS coordinates route of a moving vehicle. Every 10 checkpoints, new direction signals are updated, and the user is given directions in real-time. The arrow shaped PB pattern turns on for 2 seconds and turns off for 1 second for every real-time coordinate value. Go and Stop PB patterns do not blink. The video is played at 2x speed.
